# Detection of Phosphine Resistance in Field Populations of Four Key Stored-Grain Insect Pests in Pakistan

**DOI:** 10.3390/insects12040288

**Published:** 2021-03-26

**Authors:** Waqas Wakil, Nickolas G. Kavallieratos, Muhammad Usman, Sehrish Gulzar, Hamadttu A. F. El-Shafie

**Affiliations:** 1 Department of Entomology, University of Agriculture, Faisalabad 38040, Pakistan; usmanbhattiuaf@gmail.com (M.U.); sehrishgulzar41@gmail.com (S.G.); 2 Laboratory of Agricultural Zoology and Entomology, Department of Crop Science, Agricultural University of Athens, 75 Iera Odos str., 11855 Athens, Greece; 3 Date Palm Research Center of Excellence, King Faisal University, Hofuf, Al-Ahsa 31982, Saudi Arabia; elshafie62@yahoo.com

**Keywords:** lesser grain borer, granary weevil, red flour beetle, khapra beetle, phosphine, resistance level

## Abstract

**Simple Summary:**

The resistance of coleopteran stored-product pests to phosphine fumigation is becoming a global phenomenon. However, there is limited literature available and a lack of knowledge on this issue in Pakistan. Thus, in the current study, we estimated the status of phosphine resistance among ten populations of *Rhyzopertha dominica* (F.) (Coleoptera: Bostrychidae), *Sitophilus granarius* (L.) (Coleoptera: Curculionidae), *Tribolium castaneum* (Herbst) (Coleoptera: Tenebrionidae) and *Trogoderma granarium* Everts (Coleoptera: Dermestidae), originated from different regions of Pakistan. Laboratory-susceptible populations of all insect species were also considered in the tests. Concentration–response bioassays were conducted for each insect species. All of the tested populations of each species were found to be resistant to phosphine. However, they varied with regard to their levels of resistance. Overall, *R. dominica* exhibited the highest resistance level, followed by *T. castaneum*, *T. granarium* and *S. granarius*. Although phosphine is effective against several stored-product pests, the development of resistance may lead to failures in its application in Pakistan.

**Abstract:**

In Pakistan, the control of stored-product insect pests mainly relies on the use of phosphine gas along with other control tactics. The aim of this study was to determine the level of phosphine resistance among ten differently located populations of the lesser grain borer, *Rhyzopertha dominica* (F.) (Coleoptera: Bostrychidae), the granary weevil, *Sitophilus granarius* (L.) (Coleoptera: Curculionidae), the red flour beetle, *Tribolium castaneum* (Herbst) (Coleoptera: Tenebrionidae) and the khapra beetle, *Trogoderma granarium* Everts (Coleoptera: Dermestidae). Laboratory-susceptible populations of all insect species were also considered in the experiments. Concentration–response bioassays were conducted for each species. All of the tested populations (10 out of 10) of each species were found to be resistant to phosphine, but varied in their level of resistance. Probit analysis estimated LC_50_ at 2.85, 1.90, 2.54 and 2.01 ppm for laboratory-susceptible populations of *R. dominica*, *S. granarius*, *T. castaneum* and *T. granarium*, respectively. Against *R. dominica*, the highest and lowest resistance levels were observed in the Rahim Yar Khan (LC_50_ at 360.90 ppm) and Rawalpindi (LC_50_ at 210.98 ppm) populations, respectively. These resistant populations were 126.67- and 74.02-fold more resistant than the laboratory population. The Multan and Lahore populations of *S. granarius* exhibited the maximum (LC_50_ at 122.81 ppm) and minimum (LC_50_ at 45.96 ppm) resistance levels, respectively, i.e., they were 64.63- and 24.18-fold more resistant than the laboratory population. The Layyah population of *T. castaneum* showed the maximum resistance level (LC_50_ at 305.89 ppm) while the lowest was observed in the Lahore population (LC_50_ at 186.52 ppm), corresponding to 120.42- and 73.43-fold more resistant than the laboratory population, respectively. Regarding *T. granarium*, the Layyah population showed the maximum resistance level (LC_50_ at 169.99 ppm) while the Lahore population showed the minimum resistance (LC_50_ at 74.50 ppm), i.e., they were 84.57- and 37.06-fold more resistant than the laboratory population, respectively. Overall, *R. dominica* presented the highest resistance level, followed by *T. castaneum*, *T. granarium* and *S. granarius*. The current study suggests that the application of phosphine may not be an adequate control strategy for the management of the above tested insect pests in Pakistan.

## 1. Introduction

Control tactics applied in grain commodities before storage include the removal of grain debris and the application of insecticides [1]. Although synthetic insecticides act as efficient management tools that provide quick control of insect pests, they can have negative impact on humans’ health and the environment [2,3]. The overuse or improper use of insecticides may trigger the development of resistance to insecticides [4]. Specifically, due to heavy exposure to chemical insecticides, stored-grain insects have developed resistance to major classes of insecticides, e.g., pyrethroids and organophosphates [5,6,7,8]. Resistance to certain chemical insecticides, such as fenitrothion, pirimiphos-methyl and malathion, has been investigated in the lesser grain borer, *Rhyzopertha dominica* (F.) (Coleoptera: Bostrychidae), the rice weevil, *Sitophilus oryzae* (L.) (Coleoptera: Curculionidae), the red flour beetle, *Tribolium castaneum* (Herbst) (Coleoptera: Tenebrionidae) and the maize weevil, *Sitophilus zeamais* Motschulsky (Coleoptera: Curculionidae) [9,10]. Cases of resistance to pyrethroids have been recorded in the last two decades in *S. zeamais* [11,12,13,14]. The extensive spread of resistant populations of *S. zeamais* through grain trade within Brazil has been identified as the main issue leading to the development of insecticidal resistance [12,14]. Perez Medoza [15] also observed resistance to permethrin and deltamethrin in Mexican strains of *S. oryzae*. In Australia, the saw-toothed grain beetle, *Oryzaephilus surinamensis* (L.) (Coleoptera: Silvanidae), was found to be resistant to frequently used insecticides (e.g., chlorpyrifos-methyl, pirimiphos-methyl and fenitrothion) [16].

Fumigation with phosphine is widely used as an important method of protection of stored commodities against different insect pests. It is inexpensive, has fewer residual effects than contact insecticides, is easily applicable and represents an ideal fumigant gas that can be applied to a wide range of stored commodities and structures (e.g., warehouses, silos, bag stacks, bunkers, cereal mills and ships during transport) [17,18,19,20,21]. Additionally, phosphine has been globally trusted as a food-safe and residue-free treatment for stored grains [22]. However, the repeated use of this fumigant in storage facilities has led to the development of phosphine resistance in major stored-grain insect species [23].

During the 1970s, Champ and Dyte [24] conducted the first global survey related to phosphine resistance and documented that several main stored-grain insect pests exhibited phosphine resistance across different countries [23]. Previous studies have documented the absence, and presence, of phosphine resistance in populations of pests and revealed additional information about the existence of two phenotypes of resistance (strong and weak) in insect individuals [23,25,26,27,28]. The weak and strong resistance phenotypes are associated with 10- to 50-fold and ≥100-fold higher concentrations of phosphine, respectively, than the concentrations reported to be needed to suppress susceptible insects in the cases of the rusty grain beetle, *Cryptolestes ferrugineus* (Stephens) (Coleoptera: Laemophloeidae), *R. dominica*, *S. oryzae* and *T. castaneum* [22,29,30]. Recent studies confirmed the high incidence of phosphine resistance in major stored-grain pests at various locations around the world [25,26,27,31,32,33]. Opit et al. [26] determined that the resistance frequencies in *T. castaneum* and *R. dominica* were considerably higher than those found twenty years earlier at the same sites studied by Zettler and Cuperus [34]. More recent studies in North America reported that almost half of 25 studied *T. castaneum* populations exhibited phosphine resistance [35], while 4 out of 14 populations of adults and 9 out of 14 populations of eggs of *O. surinamensis* were also resistant to this fumigant [33].

*Rhyzopertha dominica*, the granary weevil, *Sitophilus granarius* (L.) (Coleoptera: Curculionidae) and *T. castaneum* cause considerable damage to numerous types of stored commodities in food warehouses, cereal-processing facilities and retail stores worldwide [36,37,38,39,40]. The khapra beetle, *Trogoderma granarium* Everts (Coleoptera: Dermestidae), is an extremely dangerous invasive pest of stored products of plant and animal origin [41,42,43,44]. It exists in Asia, Europe and Africa, but has also been intercepted in the USA and Australia [45,46]. Currently, it is categorized as a quarantine pest in Belarus, Canada, Mexico, Morocco, New Zealand, the USA and other countries [46]. *Rhyzopertha dominica*, *S. granarius* and *T. granarium* are primary feeders, i.e., they can easily damage whole kernels, while *T. castaneum* is a secondary feeder, i.e., it mostly prefers damaged, either mechanically or by the activity of primary pests, kernels [36]. *Trogoderma granarium* larvae accelerate the damage of sound kernels when 10–25% cracked kernels are present [47]. Despite the fact that the aforementioned pests are present in Pakistan [48,49,50], there is limited knowledge regarding their resistance to phosphine. For example, Ahmedani et al. [51] assessed two populations of *T. granarium* from just one district of Pakistan to detect their level of phosphine resistance. Therefore, the objective of the current study was to determine the level of phosphine resistance among differently geographically distributed populations of *R. dominica*, *S. granarius*, *T. castaneum* and *T. granarium* in Pakistan.

## 2. Materials and Methods

### 2.1. Test Insect Populations

**Figure 1:**
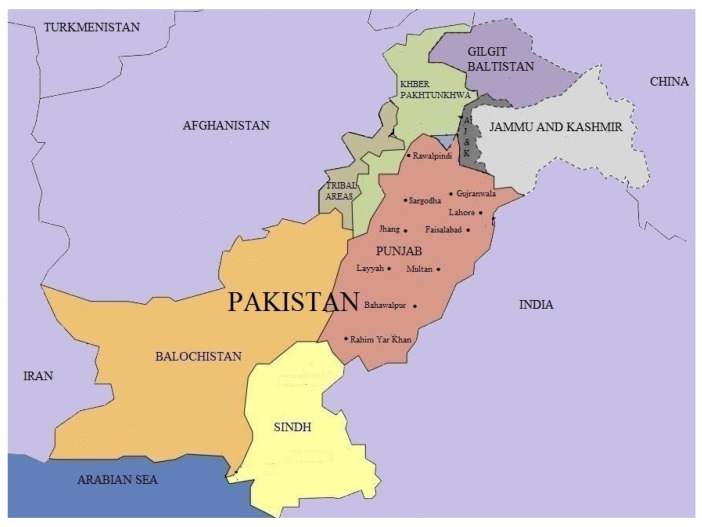
Map of Pakistan showing the regions of origin for the ten sampled populations of *Rhyzopertha dominica, Sitophilus granarius, Tribolium castaneum* and *Trogoderma granarium* tested for phosphine resistance.

Wheat-grain samples were collected from wheat-storage structures of ten different geographical regions of Pakistan, i.e., Sargodha, Multan, Faisalabad, Bahawalpur, Rawalpindi, Layyah, Lahore, Rahim Yar Khan, Jhang and Gujranwala (Figure 1). Six storage facilities were sampled per area. Within each storage facility, quantities of 500 g of wheat were taken from 5 different points. The wheat samples were mixed to make a new sample of 2.5 kg that was put inside a zip lock bag and transferred to the laboratory. The samples were sieved to separate the insects [52]. The collected *R. dominica*, *S. granarius*, *T. granarium* and *T. castaneum* adults were identified. The numbers of the collected insects ranged between 45 and 230 individuals per species and were used to start cultures. All species were cultured for at least seven generations in order to produce enough individuals for the experiments [26]. The laboratory strains of these insect pests were obtained from the Microbial Control Laboratory in the Department of Entomology, University of Agriculture, Faisalabad, Pakistan. The laboratory population had been reared for more than 10 years without being exposed to any chemical insecticides, including phosphine. *Sitophilus granarius* and *R. dominica* were reared on wheat at 25 °C, in 65% relative humidity, in complete darkness. *Trogoderma granarium* was maintained on wheat at 30 °C, in 65% relative humidity, in complete darkness. *Tribolium castaneum* was cultured on wheat flour with 5% brewer’s yeast at 25 °C, in 65% relative humidity, in complete darkness.

### 2.2. Phosphine Gas Generation

The phosphine gas was generated using the Food and Agriculture Organization of the United Nations (FAO) protocol [53]. The apparatus used for the phosphine generation included a beaker with 5 L capacity, an aluminum phosphide tablet, a collection tube (cylinder), an inverted funnel and muslin cloth. One side of the collection tube was sealed with an airtight rubber stopper that was filled with a 5% solution of H_2_SO_4_. Additionally, half of the beaker was filled with a 5% solution of H_2_SO_4_. The collection tube was carefully placed on the beaker, over the inverted funnel, without any loss of H_2_SO_4_ from the collecting tube. Before the generation of phosphine gas, all of the air was removed from the collecting tube using a syringe. The aluminum phosphide tablet was carefully wrapped with muslin cloth and put under the inverted funnel. The phosphine gas was collected in the collection tube on the underside of the inverted funnel. As the funnel filled with gas, the solution level of H_2_SO_4_ decreased. When the collection tube was filled with gas, a volume of 5 mL was taken with an airtight syringe and injected into the sealed desiccators. Then, 50 mL of gas were taken from the desiccators and injected into a phosphine meter to measure its concentration (Silo Check Phosphine Monitor, The Canary Company Pty. Ltd., Lane Cove, NSW, Australia).

### 2.3. Bioassays

For monitoring of resistance among different stored-grain insect pests, FAO method No. 16 [53] was followed. Adult *R. dominica*, *S. granarius* or *T. castaneum* (less than two weeks old) and adult *T. granarium* (less than 24 h old), of mixed-sex ratios, were used in the tests. From each field and laboratory insect population, 50 adult individuals were separately put in 4 glass vials (1.2 cm in diameter by 4.5 cm in height). The four glass vials (with one vial representing one species from one location) were grouped together with a rubber band and similar vials were prepared for the rest of the nine populations and placed in a 3.8 L glass jar prior to the introduction of phosphine gas (50 individuals of each species per vial × 4 species (= 200 adults per location) × 10 locations = 2000 individuals per jar). Seven concentrations (50, 100, 200, 300, 400, 500 and 600 ppm) of phosphine were used for the field populations and each concentration within one jar represents one treatment. There were 40 vials in one jar and a total seven jars for seven concentrations. Each insect species of the laboratory populations was exposed to seven (1, 2, 3, 6, 9, 12 and 15 ppm) phosphine concentrations. Thus, there were four vials in a single jar. Lower concentrations of phosphine were tested in the case of laboratory populations since they are highly susceptible to chemical insecticides. For each location, the same number of adults was placed inside vials in a single jar, without any treatment, to serve as controls. These vials were covered with muslin cloth and wrapped at the top with a rubber band. The muslin cloth promoted the entry of phosphine gas into the vials and also restricted the escape of insects. A small quantity of diet (0.5 g wheat) was added into each vial. The airtight jar served as a fumigation chamber with a metal screw lid at the center, equipped with a port connected to a rubber injection septum for the entrance and sampling of phosphine gas. Before the lid was screwed onto the jar, a rubber gasket was fitted inside it and a thin layer of vacuum grease was applied to the seal between the metal lid and the top of the edges of the jars to ensure the chamber was gas-tight. The gas was introduced into the jars through the rubber septum with the help of a gas-tight syringe, after removing an equal amount of air with the syringe. The maintenance of a relative humidity of up to 70% was achieved via the addition of two drops of water inside the jar. The jars were maintained inside a chamber at 25 °C for the bioassay. Twenty hours later, all vials were removed from the jars and were kept at 25 °C, in 70% relative humidity. Seven days post-application, all insects in the vials were counted and classified as live or dead [54]. Dead individuals were placed inside Petri dishes lined with filter paper, which was previously moistened with 0.5 mL of distilled water [26]. These individuals were inspected again after 24 h for possible recoveries. Each treatment (phosphine concentration) included 21 jars (7 treatment jars × 3 replications). The whole experiment was conducted twice by preparing new vials, wheat diet and insects each time (six replications totally).

### 2.4. Statistical Analyses

Mortality in controls was corrected using Abbott’s [55] formula, which was <5% for all species [28,54]. Data were analyzed using PROC PROBIT from SAS version 9.1 [56]. Lethal concentrations including LC_50_ and 95% fiducial limits were determined for each tested population from each location. Resistance ratios (RRs) were assessed by dividing the LC_50_ values of tested insecticides by their respective susceptible population.

## 3. Results

### 3.1. Resistance in R. dominica

The current study revealed that all tested populations collected from different geographical regions were found to be resistant to phosphine with reference to the laboratory population. However, the tested populations exhibited variable levels of resistance to phosphine. Probit analysis estimated that the LC_50_ for the laboratory population was 2.85 ppm. The Rahim Yar Khan population was the most resistant with LC_50_ at 360.90 ppm, i.e., it was 126.67-fold more resistant than the laboratory population. The population with the lowest resistance was obtained from Rawalpindi with LC_50_ at 210.98 ppm, i.e., it was 74.02-fold more resistant when compared with the laboratory population. For all other cases, LC_50_ ranged between 234.05 and 333.37, i.e., an 82.12- and 116.97-fold higher resistance than the laboratory population, in Lahore and Multan, respectively (Table 1).

### 3.2. Resistance in S. granarius

The populations of *S. granarius* that were collected from different geographical regions exhibited resistance to phosphine with reference to the laboratory-susceptible population. Although all of the test populations were found to be resistant to phosphine, they showed variable levels of resistance. Probit analysis evaluated the LC_50_ for the laboratory population at 1.90 ppm. The Multan population was the most resistant population with LC_50_ at 122.81 ppm, i.e., it was 64.63-fold more resistant when compared with the laboratory population, followed by the Jhang, Layyah and Bahawalpur populations, having LC_50_ values of 119.37, 107.29 and 93.72 ppm, i.e., they were 62.82-, 56.46- and 49.32-fold more resistant than the laboratory population, respectively (Table 2).

### 3.3. Resistance in T. castaneum

The tested *T. castaneum* populations were resistant to phosphine in relation to the laboratory population. Probit analysis revealed that the LC_50_ was 2.54 ppm for the laboratory population. Of the different populations, those from Layyah and Lahore showed the highest and lowest resistance levels with LC_50_ values of 305.89 and 186.52 ppm, i.e., they were 120.42- and 73.43-fold more resistant when compared with the laboratory population, respectively. The LC_50_ levels of all other tested populations ranged between 202.99 (Faisalabad) and 275.91 ppm (Jhang), i.e., between 79.91- and 108.62-fold more resistant compared with the laboratory population (Table 3).

### 3.4. Resistance in T. granarium

As in the previous species, all examined populations of *T. granarium* were found to be resistant to phosphine in relation to the laboratory-susceptible population. Probit analysis revealed that the LC_50_ for the laboratory population was 2.01 ppm. The Layyah *T. granarium* population was the most resistant, having LC_50_ at 169.99 ppm, i.e., it was 84.57-fold more resistant than the laboratory population, followed by the Jhang, Bahawalpur and Multan populations, which had LC_50_ values of 145.23, 131.11 and 127.21 ppm, i.e., they were 72.25-, 65.22- and 63.28-fold more resistant than the laboratory population, respectively (Table 4).

## 4. Discussion

Knowledge of the resistance status among different insect pests is an important issue because it influences the community to alternate the use of chemical insecticides with others that demonstrate a different mode of action [23]. During the present study, 10 out of 10 populations of *R. dominica*, *T. castaneum*, *S. granarius* and *T. granarium*, originating from different geographical localities, were found to be resistant to phosphine. In previous studies, Alam et al. [57] and Ahmad et al. [58] reported phosphine resistance in *T. castaneum*, *R. dominica*, *S. oryzae* and *T. granarium* populations collected from two remote areas of Pakistan. Surprisingly, both publications reported almost identical data for all tested insect populations. Therefore, the results of both studies should be taken into consideration with extreme caution. However our study reports phosphine resistance in insect pests from numerous areas in Pakistan, where there is no published literature available. We also report phosphine resistance in *S. granarius* for the first time in the country. In the USA, 4 out of 11 populations of *T. castaneum* collected from California were found to be resistant to phosphine, with resistance frequencies ranging between 42 and 100% [59]. Opit et al. [26] determined that eight out of nine (89%) and five out of five (100%) populations of *T. castaneum* and *R. dominica* from ten different counties of Oklahoma were found to be resistant to phosphine. Cato et al. [35] found that 12 out of 25 populations of *T. castaneum* collected from the USA and Canada were resistant to phosphine, with resistance frequencies ranging from 2–100%. In Brazil, phosphine resistance was also recorded in all 22 (100%) of the different populations of *S. zeamais* studied [60]. The occurrence of phosphine resistance might be due to the presence of a resistance gene in the population, and also by the selection pressure from phosphine application. The flight activity of adults and the shipment of commodities that contained insect pests with the resistant gene are held responsible for gene flow, increasing phosphine resistance [61].

Phosphine resistance at least 100 times higher than susceptible populations has been recorded in different continents including Africa, Asia, Australia and South America [25,62,63,64,65,66,67,68,69,70,71]. In Pakistan, Ahmedani et al. [51] recorded a 2.54–3.98-fold increase in resistance level in two populations of *T. granarium*, collected from two remote areas of the Rawalpindi district, compared with a susceptible population. The authors concluded that the historical application of phosphine gas in these locations could not explain the low level of phosphine resistance. However, in the present study, the observed resistance to phosphine is 84.57-fold higher in *T. granarium*. Although the sampled locations in our study are different from the aforementioned study, resistance to phosphine has increased with time. The trade of infested grains among areas that host storage facilities results in the spread of resistance genes among insects’ populations, and consequently leads to the increased resistance in an area [35,61]. Opit et al. [26] reported LC_99_ at 377.49 and 3430.8 ppm for resistant *T. castaneum* and *R. dominica* populations, respectively, from Oklahoma, which was much higher compared with Zettler and Cuperus [34], who determined their resistance levels 21 years before (i.e., 9.2 and 6.6 ppm respectively). The different level of resistance ratios among different populations of the same species, as observed in this study, might be due to their genetic diversity [72,73] and distinct geographical areas [35]. The elevated frequency of phosphine applications in a given area is associated with low genetic diversities of stored-product coleopterans, compared with areas with less frequent applications of phosphine, due to the suppression of low frequency haplotypes [74,75].

In Pakistan, different types of storage structures, including conventional house-type warehouses (“godowns”), hexagonal bins, binishells, bunkers, concrete/steel silos and temporary open storage (“ganjis”), are used to store wheat commodities. Conventional warehouses hold almost 70% of stored grains, followed by binishells at 13%, hexagonal bins at 7%, bunkers at 6% and concrete/steel silos at 4%. More than 60% of wheat is stored in bags on outdoor mats and covered by polythene [76]. Phosphine is a common fumigant for treating grains and the overuse of a single fumigant, as well as underdosing, can be a cause of the emergence of resistance in stored-grain insect pests. The most resistant insect populations originated from the southern part of Pakistan (e.g., Layyah, Multan and Rahim Yar Khan) where very hot summers prevail. Nayak et al. [77] reported that warm temperatures favor the elevated population growth of insect pests that are associated with the appearance of strongly resistant populations. Furthermore, a number of additional factors might be responsible for the increase in resistance to phosphine, e.g., leaky storage structures that lead to underdosing of phosphine, no phosphine-concentration monitoring during fumigation, exclusive reliance upon a single type of fumigation, little or no focus on integrated pest management at the farm level and insect movement through the commodity trade [12,25,33,78]. The mechanism involved in phosphine resistance is that of resistant individuals receiving lower or overused phosphine gas, compared with susceptible ones [4,79,80,81,82].

Our findings determined different levels of phosphine resistance, weak and strong, among the tested species. Four populations (i.e., Lahore, Gujranwala, Rawalpindi and Rahim Yar Khan) of *S. granarius* presented weaker resistance compared with the other insect pest populations. In the current study, the most resistant population of *R. dominica* had LC_50_ at 360.90 ppm after 20 h, which equate to 7,218 ppm per h to kill the insect population. This gas concentration should be maintained under airtight conditions. However, it is not possible to retain such high concentrations of phosphine due to leaky and poor storage conditions in Pakistan. Thus, it is difficult to attain the required level of phosphine concentration, leading to resistance in insect pests. This is an issue that encourages the replacement of phosphine with alternative approaches for the sustainable management of stored-grain insect pests in Pakistan. For the first time Collins et al. [83] described different levels of phosphine resistance in insects and detected two levels of resistance in *R. dominica*, i.e., weak resistance, corresponding to a 23-fold increase, and strong resistance, corresponding to >100-fold increase. Similarly to our findings, Afful et al. [23] reported weak and strong resistance among populations of *R. dominica* originating from North America. The authors concluded that the low resistant population ranged between 5.2- to 9.5-fold higher resistance while the strong resistant population exhibited 100.2- to 595.9-fold higher resistance when compared with the susceptible population. Schlipalius et al. [72] elaborated that insects have a distinct weak resistant phenotype which is homozygous to the resistance alleles on gene locus (usually stated as rph1). However, the strong resistant phenotype requires the insect to be homozygous at rph1 as well as at the second locus for resistance (referred to as rph2), and the two loci work synergistically to indicate the strong resistant phenotype.

The phosphine concentrations obtained during our study were much higher than those recommended against *R. dominica*, *T. castaneum*, *T. granarium* and *S. granarius*. For instance, Opit et al. [26] found that LC_99_ ranged between 377 and 3431 ppm against *T. castaneum* and *R. dominica* 72 h post-exposure period. Additionally, Gautam et al. [20] determined LC_99_ at 1030.7 ppm for killing the eggs of the most resistant population of *O. surinamensis* from the USA, compared with the 28.4 ppm required to kill the eggs of the susceptible laboratory population after 72 h of exposure interval. Considering the aforementioned issues, there are some management approaches that need to be adopted to overcome the phosphine resistance. An important step is to construct a “national-resistance-monitoring program” [61] by recording the occurrence of resistance in different stored-product insect species in different regions of Pakistan, according to Ahmedani et al. [51] and the current study. Next, the recommended phosphine concentrations should be revised on the basis of laboratory and field trials [67,69]. Other management tactics involve eradication of the resistant populations through extended exposure periods to phosphine, an increase in the concentration of phosphine, proper sealing of storage facilities, the replacement of old phosphine application equipment, enhancement of grain turning, application of grain protectants, sanitization of storage facilities, implementation of residual insecticides, monitoring of populations of pests through inspection and sampling, as well as the use of alternative gases (e.g., sulfuryl fluoride) [68,69,71].

## 5. Conclusions

Our study is the first comprehensive report on the detection of the level of phosphine resistance in 10 different populations of *R. dominica*, *T. castaneum*, *T. granarium* and *S. granarius* collected from geographically distinct districts of Pakistan. Our findings revealed that all tested species and populations are resistant to phosphine fumigation—therefore alternative management strategies should be pursued. We detected variable levels of phosphine resistance among the tested insect pests. The frequency and prevalence of resistant individuals indicate widespread insufficiency of fumigation practices and a high incidence of improperly sealed storage facilities. Thus, we recommend improvements in storage practices and structures, as well as the implementation of resistance-management programs. Furthermore, plans at the national level should be elaborated in order to moderate the resistance to phosphine—retaining this fumigant as a feasible stored-grain pest-management tool.

## Figures and Tables

**Table 1:** Probit analysis of mortality for 10 field-collected populations and a laboratory-susceptible population of *Rhyzopertha dominica* adults after 20 h of exposure to phosphine. In all cases DF = 5.

Region	LC_50_ (ppm) 95% FL	Slope	Chi-Square	*p*	Intercept	RR ^a^
Sargodha	236.32 (163.06–259.16)	2.35 ± 0.99	78.38	<0.01	−6.79 ± 1.58	82.91
Multan	333.37 (112.01–415.37)	3.02 ± 0.92	36.57	<0.01	−7.63 ± 1.45	116.97
Faisalabad	257.99 (23.21–342.38)	2.42 ± 0.76	71.85	<0.01	−21.37 ± 1.98	90.52
Bahawalpur	295.83 (215.23–376.28)	2.89 ± 1.47	53.86	<0.01	−7.15 ± 1.90	103.80
Rawalpindi	210.98 (163.06–259.16)	2.28 ± 0.25	26.76	<0.01	−5.31 ± 0.62	74.02
Layyah	287.72 (173.37–357.61)	2.73 ± 0.86	62.93	<0.01	−6.72 ± 1.82	100.95
Lahore	234.05 (186.14–309.39)	2.96 ± 0.13	57.91	<0.01	−7.01 ± 1.96	82.12
Rahim Yar Khan	360.90 (98.92–441.17)	4.19 ± 0.58	46.22	<0.01	−11.09 ± 1.12	126.67
Jhang	315.36 (206.20–381.26)	2.90 ± 1.19	47.00	<0.01	−7.25 ± 1.16	110.65
Gujranwala	279.88 (171.28–416.52)	3.31 ± 0.56	78.31	<0.01	−8.10 ± 1.21	98.20
Laboratory	2.85 (2.62–3.07)	2.48 ± 0.10	7.44	0.18	−1.13 ± 0.07	-

LC: lethal concentration; FL: fiducial limits; RR: resistance ratio; ^a^ RR = LC50-resistant population/LC50-susceptible population.

**Table 2:** Probit analysis of mortality for 10 field-collected populations and a laboratory-susceptible population of *Sitophilus granarius* adults after 20 h of exposure to phosphine. In all cases DF = 5.

Region	LC_50_ (ppm) 95% FL	Slope	Chi-Square	*p*	Intercept	RR ^a^
Sargodha	86.60 (69.64–103.03)	2.20 ± 0.15	11.05	0.05	−4.26 ± 0.34	45.57
Multan	122.81 (89.15–155.13)	2.31 ± 0.24	26.55	<0.01	−4.82 ± 0.56	64.63
Faisalabad	71.26 (54.62–87.26)	2.10 ± 0.15	11.85	0.03	−3.90 ± 0.35	37.50
Bahawalpur	93.72 (75.20–111.68)	2.15 ± 0.14	11.29	0.04	−4.25 ± 0.34	49.32
Rawalpindi	48.58 (27.28–68.61)	2.03 ± 0.25	25.18	<0.01	−3.43 ± 0.54	25.56
Layyah	107.29 (81.23–132.49)	2.33 ± 0.21	21.74	<0.01	−4.73 ± 0.49	56.46
Lahore	45.96 (28.55–62.43)	2.06 ± 0.21	18.07	<0.01	−3.42 ± 0.47	24.18
Rahim Yar Khan	63.17 (44.80–80.62)	2.19 ± 0.20	16.79	<0.01	−3.95 ± 0.442	33.24
Jhang	119.37 (84.69–152.65)	2.25 ± 0.24	28.55	<0.01	−4.68 ± 0.57	62.82
Gujranwala	66.75 (60.94–80.96)	2.11 ± 0.14	9.92	0.07	−3.85 ± 0.32	35.13
Laboratory	1.90 (1.51–2.28)	2.21 ± 0.16	12.23	0.03	−0.61 ± 0.10	-

LC: lethal concentration; FL: fiducial limits; RR: resistance ratio; ^a^ RR = LC50-resistant population/LC50-susceptible population.

**Table 3:** Probit analysis of mortality for 10 field-collected populations and a laboratory-susceptible population of *Tribolium castaneum* adults after 20 h of exposure to phosphine. In all cases DF = 5.

Region	LC_50_ (ppm) 95% FL	Slope	Chi-Square	*p*	Intercept	RR ^a^
Sargodha	223.06 (156.78–298.67)	3.79 ± 0.54	79.47	<0.01	−8.91 ± 1.97	87.81
Multan	261.00 (24.62–343.96)	3.38 ± 1.10	54.30	<0.01	−8.16 ± 1.88	102.75
Faisalabad	202.99 (113.75–260.90)	2.75 ± 0.73	89.89	<0.01	−6.36 ±1.51	79.91
Bahawalpur	245.43 (66.41–341.11)	3.17 ± 0.99	48.54	<0.01	−7.59 ± 1.71	96.62
Rawalpindi	234.63 (141.27–289.47)	3.26 ± 1.29	67.19	<0.01	−7.41± 1.36	92.37
Layyah	305.89 (131.04–374.82)	3.73 ± 1.04	41.34	<0.01	−9.27 ± 1.72	120.42
Lahore	186.52 (110.43–243.79)	2.64 ± 0.43	32.91	<0.01	−6.00 ± 1.10	73.43
Rahim Yar Khan	256.60 (172.34–345.16)	3.50 ± 1.48	67.50	<0.01	−8.45 ± 2.87	101.02
Jhang	275.91 (74.74–355.02)	3.24 ± 0.95	47.26	<0.01	−7.91± 2.50	108.62
Gujranwala	213.24 (189.24–265.19)	2.83 ± 0.50	86.26	<0.01	−6.61 ± 1.91	83.92
Laboratory	2.54 (2.34–2.75)	2.41 ± 0.10	6.35	0.27	−0.98 ± 0.07	-

LC: lethal concentration; FL: fiducial limits; RR: resistance ratio; ^a^ RR = LC50-resistant population/LC50-susceptible population.

**Table 4:** Probit analysis of mortality for 10 field-collected populations and a laboratory-susceptible population of *Trogoderma granarium* adults after 20 h of exposure to phosphine. In all cases DF = 5.

Region	LC_50_ (ppm) 95% FL	Slope	Chi-Square	*p*	Intercept	RR ^a^
Sargodha	86.29 (77.48–94.96)	2.15 ± 0.09	6.39	0.26	−4.16 ± 0.22	42.93
Multan	127.21 (115.25–139.05)	1.95 ± 0.09	4.48	0.48	−4.11 ± 0.22	63.28
Faisalabad	100.35 (69.68–129.76)	1.75 ± 0.18	20.01	<0.01	−3.51 ± 0.42	49.92
Bahawalpur	131.11 (100.44–161.00)	2.06 ± 0.18	18.18	<0.01	−4.36 ± 0.44	65.22
Rawalpindi	75.47 (66.65–84.12)	1.96 ± 0.09	4.17	0.52	−3.69 ± 0.22	37.54
Layyah	169.99 (127.74–212.78)	1.98 ± 0.21	25.57	<0.01	−4.42 ± 0.51	84.57
Lahore	74.50 (19.81–126.43)	1.38 ± 0.28	53.47	<0.01	−2.59 ± 0.67	37.06
Rahim Yar Khan	117.76 (99.48–135.73)	2.40 ± 0.14	11.00	0.05	−4.97 ± 0.34	58.58
Jhang	145.23 (103.79–185.91)	1.89 ± 0.21	25.01	<0.01	−4.10 ± 0.50	72.25
Gujranwala	109.19 (100.36–117.92)	2.48 ± 0.10	5.71	0.33	−5.05 ± 0.23	54.32
Laboratory	2.01 (1.82–2.21)	2.20 ± 0.18	7.81	0.16	−0.67 ± 0.17	-

LC: lethal concentration; FL: fiducial limits; RR: resistance ratio; ^a^ RR = LC50-resistant population/LC50-susceptible population.

## Data Availability

Data is contained within the article.
